# Experimental Investigations into the Mechanical, Tribological, and Corrosion Properties of Hybrid Polymer Matrix Composites Comprising Ceramic Reinforcement for Biomedical Applications

**DOI:** 10.1155/2018/9283291

**Published:** 2018-08-23

**Authors:** Mohammed Yunus, Mohammad S. Alsoufi

**Affiliations:** Department of Mechanical Engineering, College of Engineering and Islamic Architecture, Umm Al-Qura University, Al-Abdiah, Makkah 24231, Saudi Arabia

## Abstract

Hybrid polymer matrix composites (HPMC) are prominent material for the formation of biomaterial and offer various advantages such as low cost, high strength, and the fact that they are easy to manufacture. However, they are associated with low mechanical (low hardness) and tribological properties (high wear rate). The average hip joint load fluctuates between three to five times of the body weight during jumping and jogging and depends on various actions relating to body positions. Alternate bone and prosthesis material plays a critical role in attaining strength as it determines the method of load transferred to the system. The material property called modulus of elasticity is an important design variable during the selection of the geometry and design methodology. The present work is demonstrated on how to improve the properties of high-density polyethylene (HDPE) substantially by the addition of bioceramic fillers such as titanium oxide (TiO_2_) and alumina (Al_2_O_3_). The volume fractions of Al_2_O_3_ and TiO_2_ are limited to 20% and 10%, respectively. Samples were fabricated as per ASTM standards using an injection moulding machine and various properties such as mechanical (tensile, flexural, and impact), tribological (hardness, wear), and corrosion including SEM, density, and fractography analysis studied. Experimental results revealed that an injection moulding process is suitable for producing defect-free mould HPMC. HPMC comprising 70% HDPE/20% Al_2_O_3_/10% TiO_2_ has proved biocompatible and a substitute for biomaterial. A substantial increase in the mechanical and tribological properties and full resistance to corrosion makes HPMC suitable for use in orthopaedic applications such as human bone replacement, bone fixation plates, hip joint replacement, bone cement, and bone graft in bone surgery.

## 1. Introduction 

Polymer and ceramic oxide metal matrix composites are seeing applications as a biomaterial because their combination acts to replace human tissues and anatomical elements to treat or improve and also medical devices for implants. The mechanical properties are important factors that determine the progress of potential biomaterial. Polyethylene is one of the readily available low-cost polymer materials and can be processed at temperatures of 150-250°C.

Polyethylene is hybrid-linked and is available in the form of linear low, low, and high-density polyethylene (LLDPE, LDPE, and HDPE), respectively. Alternate bone materials are required to fill the gap or portion of the bone missing as bone is a natural composite mainly consisting of organic as well as mineral matrices called collagen fibers (which introduce the mechanical properties of toughness and viscoelasticity) and hydroxyapatite (HAP) forms a bonding gel. However, the challenge for alternative bone materials is to maintain a balance between biological and biomechanical properties to act as a biomaterial. The hybrid linking nature of polyethylene develops a thick chain of high molecular weight leading to the branched structure to improve the mechanical properties such as impact strength, crack, creep, and abrasion resistance without much change in tensile strength and density [1]. Alumina (Al_2_O_3_) and titanium oxides (TiO_2_) are used to improve the mechanical as well as tribological properties (wear properties) of high-density polyethylene (HDPE) further [2]. Alumina is a hard-ceramic oxide (up to 1800 HV) thermodynamically stable (up to 2000°C) which is available in both ionic and covalent bonds [3]. Titanium, meanwhile, due to its outstanding characteristics of biocompatibility, has no reaction with the tissue surrounding the implant, decreases the electronic exchange process so that there is no corrosion, has no problem with cardiac and cardiovascular applications, and has an isoelectric point to maintain a pH value between 5 and 7 of the human body [4]. Both materials are biologically inert as they remain intact after implantation into human bodies and can be retained as foreign materials by accommodation in fibrous tissues in order to isolate them from a human body. There are thus no adverse reactions yet they are endured well by tissues [5].

In the literature study, mostly the work is on the biomaterials such as stainless steel (316L), titanium alloy (Ti-6AL-4V), cobalt-chromium alloy, HAP, UHMWPE, Al_2_O_3_, TiO_2_, and silicon carbide (Sic). The above materials are used for the replacement of knee, hip, and ankle joints as well as for dental implants [6]. In the present work, we study the essential properties of bone alternate or replacement material made of high-density polyethylene. This paper focuses on the study of fundamental attributes needed for other biomaterials based on hybrid polymer matrix composites to accommodate the types of bones and joints fractured [7].

Mostly alumina and zirconia-based ceramics have used bioceramics mainly due to their biocompatibility for an implant, offering a high mechanical strength with no reaction and being nontoxic to tissues along with having a blood compatibility characteristic [8]. The mechanical properties of the 12, 24, and 36% of hybrid fiber (Sisal, Jute, and Hemp) polymer composite material when compared with the femur bone strengths are seen to be increased by increasing the percentage of the fiber [9]. The tensile and compression strength of 30% of Sisal natural fiber reinforcement epoxy composite materials was found to be maximum out of the composition of 10, 20 and 30% [10]. Variation of % of fiber weight of banana fiber in glass reinforced hybrid polypropylene composites improved various mechanical properties such as tensile, flexural, impact at 10% fiber fraction [11]. Hybrid reinforced composites formed by bamboo fiber with fly ash filler prepared by hand lay-up technique produced excellent mechanical properties [12]. Coconut shell fiber reinforced hybrid composites produced by coconut shell fiber compacting epoxy resin matrix with 10 to 30% volume fraction showed increased tensile strength with increase in coconut shell fiber content [13]. Carbon fiber based hybrid polymer composite matrix with +/- 0 to 90° orientations was used as implant material and compared with mechanical properties of a femur bone [14]. Synthesis of hybrid biopolymer matrix composites uses low-density polyethylene as matrix material with reinforcing material; namely, alumina and titanium oxide showed improvement in mechanical properties [15].

An attempt has been made to develop hybrid biopolymer matrix composites using high-density poly ethylene as the matrix material with titanium oxide/titania (TiO_2_) and alumina/aluminium oxide (Al_2_O_3_) particles as the reinforcement material with varying percentages using an extrudal injection moulding machine. The different testing, namely, tensile, hardness, flexural strength, density, fractography, corrosion, and wear test, was conducted on the standard samples prepared [16]. Substantial improvements are found in the mechanical and tribological properties of the hybrid polymer matrix composite, which can be used for a variety of applications in human body bone replacement [17]. In this case, their application in orthopaedics as an implantable material in bone surgery has been considered and studied. These composite materials have found extensive use in orthopaedic applications, particularly in bone fixation plates, hip joint replacement, bone cementing, and bone graft [18].

In the literature review, most of the work has been conducted on biomaterials such as stainless steel 316L, titanium alloy (Ti-6AL-4V), cobalt-chromium alloy, hydroxyl apatite (HAP), ultra-high molecular weight polyethylene (UHMWPE), alumina (Al_2_O_3_), titanium oxide (TiO_2_), and silicon carbide (sic) as the material for replacement of knee joints, hip joints, and ankle joints as well as dental implants. In this work, we study the essential properties of bone alternate or replacement materials for bone [19, 20].

This paper highlights the study of the fundamental properties required to replace bone materials for various types of bones and joints fractured by the synthesis of biocompatible, hybrid polymer matrix composites [21]. Polymer matrix composite is a material consisting of polymer (resin) matrix combined with a fibrous reinforcing dispersed phase. Polymers make ideal matrix material; they can be processed, i.e., being fabricated more easily, processing light weight, and offering desirable mechanical properties. The reasons for the selection of these composites are their low cost, high strength, and simple manufacturing principles [22].

## 2. Materials and Methods

Three grades of commercially available polyethylene are low-density, high-density, and ultra-high molecular weight polyethylene (UHMWPE). UHMWPE produces low ductility and fractures toughened material more than those of other classes of polyethylene. However, high-density polyethylene (HDPE) materials have better packing of linear chains and high branching levels which results in their increased crystallinity and enhanced mechanical, tribological properties [23]. Thus, in the present work, HDPE in granule form (transition/softening temperature of 125°C and melt flow index of 0.22 g/min) is used as polymer matrix material and further their properties can be improved by adding metallic (TiO_2_) as a coupling agent and ceramic reinforcement (Al_2_O_3_) materials in the polymer matrix of 325 mesh size were used for the synthesis of polymer composites supplied by alumina Ceramic Manufacturers India, Gujarat, INDIA. Hence, Al_2_O_3_ and TiO_2_ of purists grade have a melting temperature of >350°C. To obtain the various levels of properties [24], different compositions are used as shown in Table 1 by varying percentage by weight of each matrix material; Al_2_O_3_ and TiO_2_ powder materials along with a surfactant (non-inphinoethoxylate) material were used for the synthesis of composite material.

### 2.1. Production of HPMC Composites

Samples were prepared using 75 tonnage vertical injection moulding machine in which raw material is injected into a mould via a hot barrel to take the inverse shape. Multiple cavity mould is preferred over the single cavity mould to save raw material and the time. Finally the compound is then taken for the specimen preparation. Standard test specimens were prepared as per the ASTM standard for tensile (ASTM D638), flexural (ASTM D790), and impact tests (ASTM D256).

### 2.2. Mechanical and Tribological Tests

Tension tests were performed according to ASTM D 638 standard using Instron UTM (Universal Testing Machine) 4302 having a load capacity ranging from 0 to 1 0000N at the crosshead speed of 0.0166 mm/Sec. Tensile test specimen has dimensions 63 mm x 9.53 mm x 3.5 mm with cross section width 9.53 mm and radius of 12.7 mm. Flexural test was conducted in accordance with ASTM D790 having dimensions of 127 mm x 13 mm x 3.5 mm using Instron UTM 3365 possessing a load cell capacity of 50000N with centre loading three-point load system. The crosshead speed of 0.05 mm/sec and span length of 70 mm were set. The impact test from 0 to 10 J at an interval of 0.0001 J on the specimen a 64 mm x 13 mm x 3.5 mm and hardness of the synthesised polymer composites were measured using shore hardness D-scale. Durometer hardness tester (Shore Instrument and MFG Co., Freeport, NY) as per the ASTM D2240 standard was used for testing hardness of samples. Pin-on-disc sliding wear testing machine [25] was used to understand the dry sliding wear characteristics of the hybrid composite specimens. After each test, the coefficient of friction (COF) and height loss was recorded. As per ASTM G99-95 standards, the pin of Ø8 × 32 mm length was used for the tribology test. The pin was cleaned with acetone and its initial mass was measured using a digital electronic balance and then held pressed against the rotating EN-32 steel disc (counter face) with a hardness of 65 HRC during the test. The tribological test was carried out with the normal load varying from 10 N to 30 N, a track diameter of 75 mm, and sliding speed of 500 rpm, and the entire test was carried out for 15 minutes duration. Further, a corrosion test was carried out on the composites as per a salt spray test according to the ASTM B117 standard.

## 3. Results and Discussion

In this section, the mechanical and tribological characteristics of HDPE /TiO_2_ /Al_2_O_3_ hybrid composites are discussed by carrying out various tests according to ASTM standards on the specimens prepared using an injection moulding machine/process. The results of tensile strength at ultimate point, flexural strength at 7.8 mm deflection, impact strength at breaking point, shore hardness D-scale, and wear and coefficient of test depict/represent the mechanical and tribological characterization of developed HPMC including the corrosion test.

### 3.1. Mechanical Properties

#### 3.1.1. Ultimate Tensile Strength

**Figure 1:**
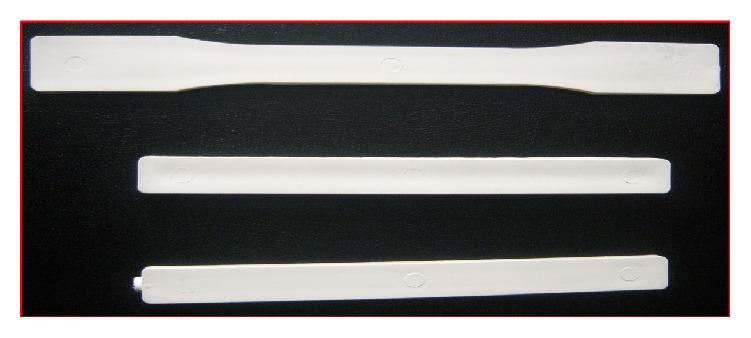
Photographs of samples prepared.

**Figure 2:**
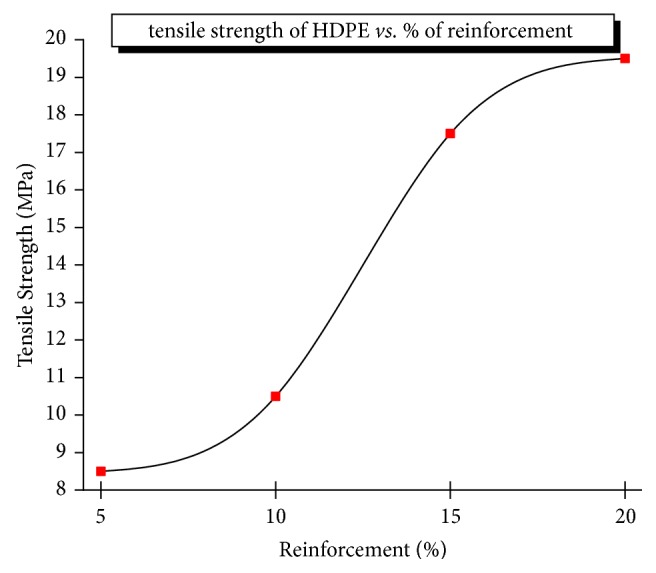
Effect of Al_2_O_3_ loading on the tensile strength of HPMC.

**Figure 3:**
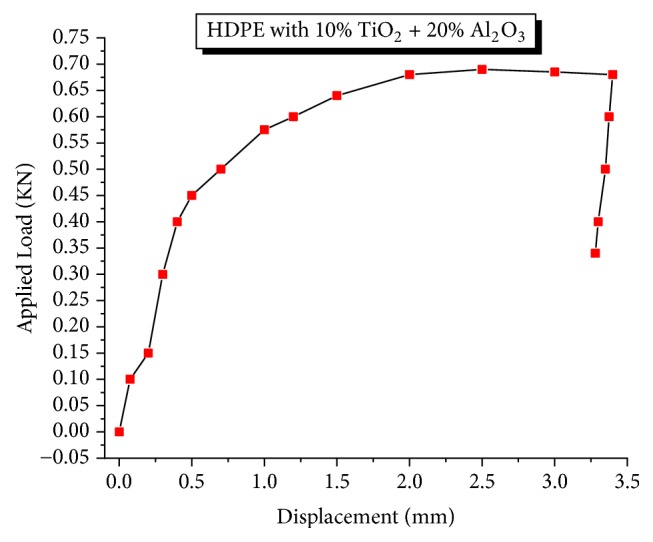
Applied load versus displacement curve of HPMC (sample 4).

The tensile specimens were tested, calculated, and plotted for the ultimate tensile strength of the composite material (refer to Figure 1). Its tensile strength increases substantially with the increase of Al_2_O_3_ (from 5 to 20% at the interval of 5%), at fixed 10% of TiO_2_ which in turn increases the load carrying capacity due to the presence of hard and stiff alumina particles in the composite material. Hence, the load carrying capacity of the composite material increases. It reaches a maximum value of 17 MPa which is 30% higher than the polyethylene with an elastic modulus of 500 MPa for the combination of 70% HDPE/20% Al_2_O_3_ /10% TiO_2_ (which remains constant) of the composite specimens as seen in the stress-strain curve of Figures 2 and 3. The elongation (ductility) of the HDPE is also increased. The presence of alumina along with TiO_2_ has increased ductility as well as little brittleness which improved both tensile strength and elongation capability. Sample 04 exhibits good yield strength strain but poor elongation due to brittleness provided by alumina reinforcements as seen in Table 2.

#### 3.1.2. Flexural Strength

**Figure 4:**
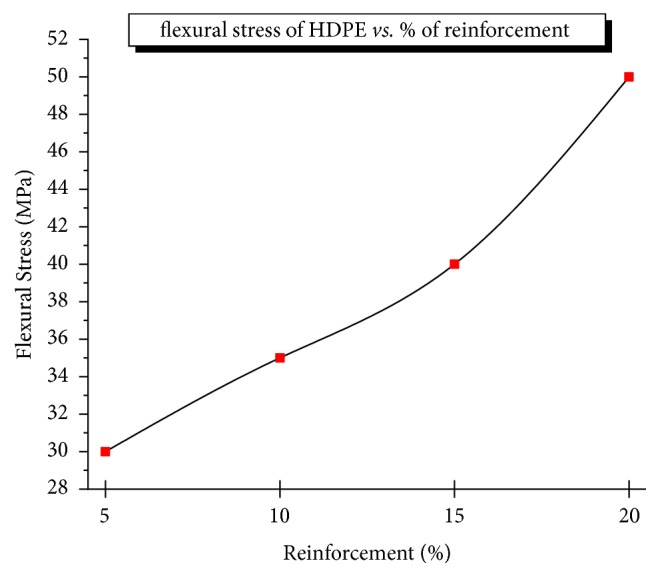
Variation of bending stress with % reinforcement.

Figure 4 shows that the variation in flexural strength of the composite specimen also increases with an increasing percentage of Al_2_O_3_ (from 5 to 20%) as they resist the deformation of the composite material. The flexural strengths of the joint were found to be about 25% higher than that of polyethylene. The plastic region of sample depends on ductility of the HDPE matrix and interparticle distance of reinforcements. At higher % Al_2_O_3_, the interparticle distance increases make matrix hard to undergo yielding, increasing the flexure strength. When the Al_2_O_3_ content was less than 5% weight, the interparticle distance reaches a minimum which can change into plastic yield to decrease the flexure strength. At very low % Al_2_O_3,_ the interparticle distance changes HPMC to behave brittle. The highest flexural strength reached a value of 50 MPa for 70% HDPE /10% TiO_2_ /20% Al_2_O_3_ and showed good bend properties. There were no visible cracks in the midsection of the composite specimen.

#### 3.1.3. Hardness

**Figure 5:**
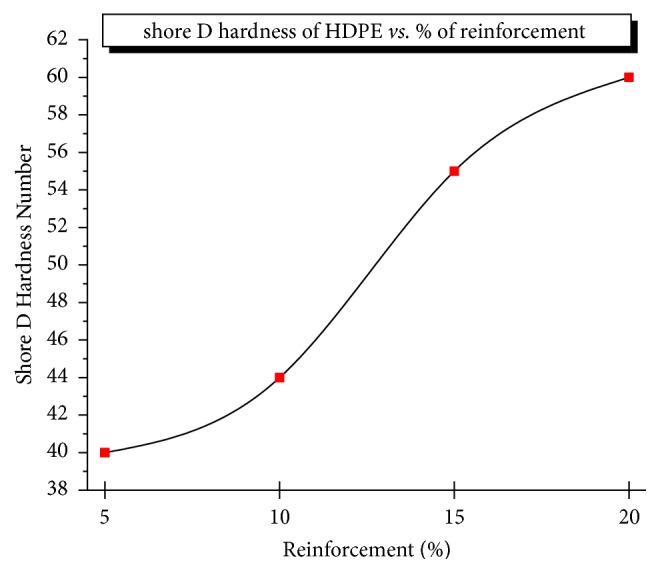
Variation of hardness with % reinforcement.

**Figure 6:**
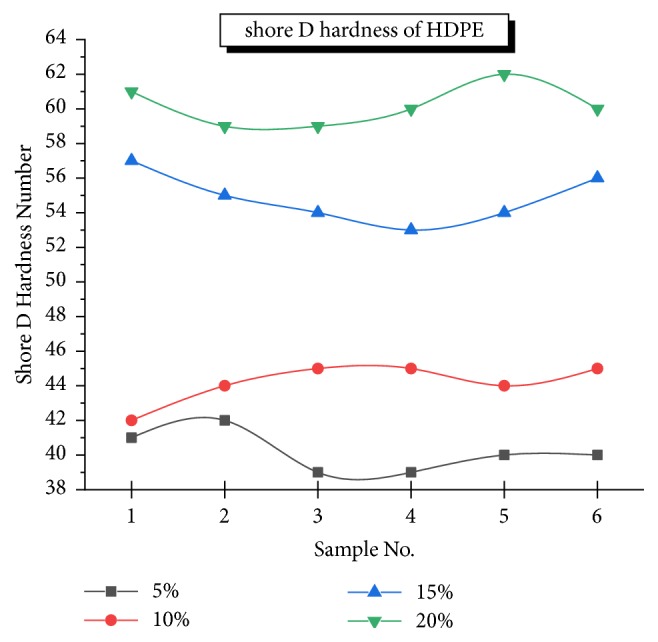
Variation of hardness with % reinforcement.

Figures 5 and 6 show the hardness profile measured and it is clearly evident that there is a substantial increase in the HDPE with an increment of Al_2_O_3_ percentage reinforcement in alumina-titania-HDPE composite compared to the unfilled system. The highest average shore D hardness number was found to be 60 measured at various sections due to uniform distribution of hard alumina particles as well as titanium oxide being bonded together and increasing the resistance to plastic deformation. The mean values of hardness against different TiO_2_ and Al_2_O_3_ of varying concentrations by weight are shown in Figure 6. The shape S indicates hardness increases rapidly but it is limited by interparticle bonding, distance, and nonuniform distribution of reinforcements. Variation of alumina and titania contributes to the hardness of HPMC.

#### 3.1.4. Density Test

**Figure 7:**
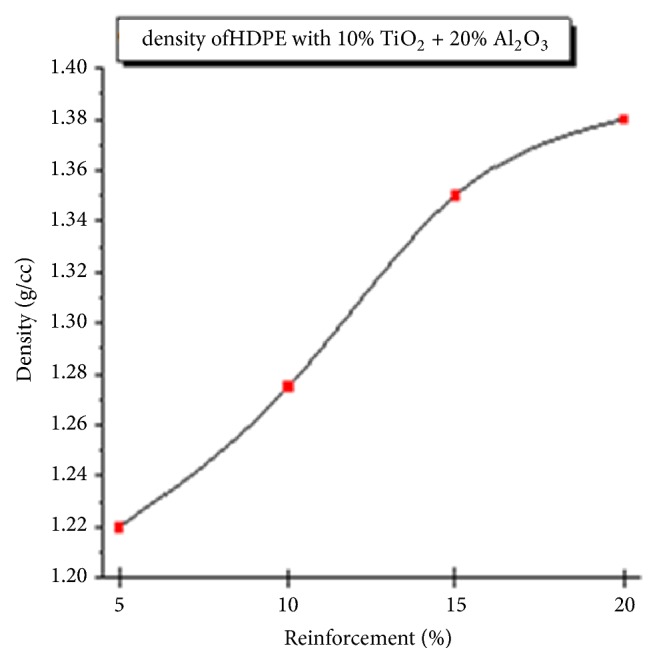
Density varying with % reinforcement.

The density of composite material depends on parent metal and constituents. Density increased with percentage of alumina and titania constituents but increasing percentage alumina contributed towards density increase due to good bonding with polyethylene as well as titania without changing the cross-linked or branched structure of the composite material. Interparticle distance, distribution of reinforcement particles, weight density difference of reinforcements and matrix polymer, and so on decide the final density. S distribution indicates density value accelerates rapidly with shallow growth during lower and higher % Al_2_O_3_ as the interparticle distance becomes too large and small, this leads to deboning.  Figure 7 shows the variation of density with the percentage of reinforcement to bring the structure to a specific weight which is necessary to use as bone or hip joint replacement material.

#### 3.1.5. Fractography Study

**Figure 8:**
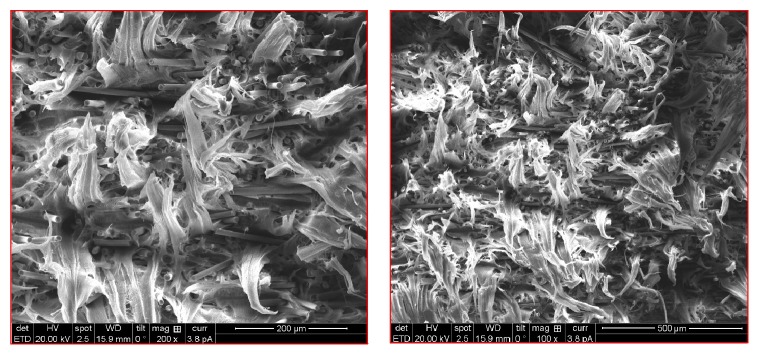
Fractured surface after tensile strength test for HDPE/20% Al_2_O_3_.

The hybrid composite HDPE/20% Al_2_O_3_/10% TiO_2_ was scanned using SEM (scanning electron microscopy) to get an image of the distribution of the reinforcing particles before the test and the fracture type after the tensile test. More details of the SEM machine procedure have been reported elsewhere [26–28].  Figure 8 shows the fractured surface observed at 200X magnification. It is clear from the SEM image that there is a homogeneous distribution of reinforcing particles in the matrix of the polymer and there are no casting defects observed. Also, there is a proper bonding between the matrix and reinforcing particles. This enhances the mechanical properties of the composite materials. Further, the image shows that the composite fails by brittle fracture. The image of the fractured surface shows a uniform composite without moulding imperfections. Beautiful populated dimples were observed at the higher magnification which resembles the brittle mode of the failure.

#### 3.1.6. Corrosion Test

As per ASTM B117, a standard corrosion test is carried out by using salt spray test involving a solution of 5% NaCl (AR Grade) with 1.41 ml volume of solution collected per hour on area of 80 Cm^2^ at a temperature of 33 to 37°C in distilled water (7.08 pH value). After cleaning with running water, no signs of corrosion or reaction were found on any of the specimens of HPMC during the observations made after a period of 24 hours using the procedure and results are tabulated in Table 3.

#### 3.1.7. Impact Strength

**Figure 9:**
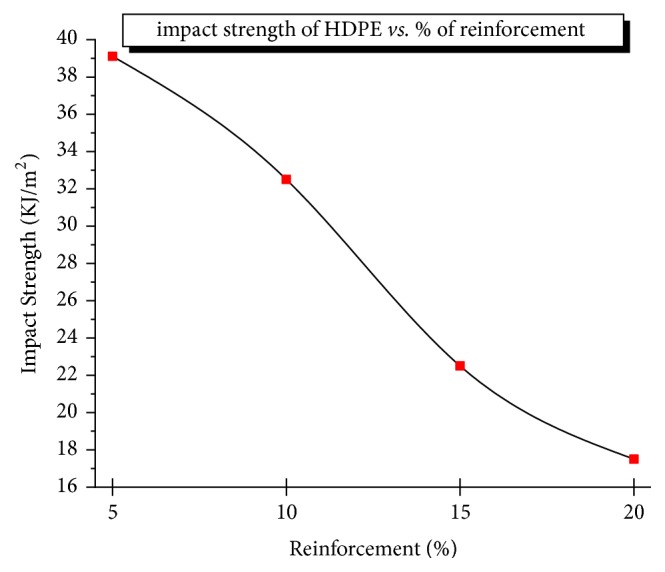
Impact strength varying with % reinforcement.

The amount of energy absorbed evaluated at the breaking point of the composites or toughness of material decreases drastically with an increasing percentage of alumina at a fixed value of TiO_2_, because of the brittle and hard nature of alumina particles which provide ductility along with titania under the static or slow rate of loading, whereas, for suddenly applied loads, bonding between alumina and titania particles breaks soon without allowing further transfer of load due to higher percentage of alumina and breaking of the bonding that exists between reinforcement constituents. In case of low percentage of alumina, load transfer happens between polyethylene and titania rather than alumina particles. Hence, the impact strength can also be increased by refining alumina particle size or intensifying bonding structure between TiO_2_ and Al_2_O_3_. The maximum impact strength obtained was 39kJ/m^2^ for HDPE/5% Al_2_O_3_/10% TiO_2_ which are depicted in Figure 9. The toughness of the alumina particles depends on ductility of the HDPE matrix and interparticle distance of reinforcements. These introduced stress concentrations lead to deboning of the filler particles and in turn void formation. The interparticle distance depends on particle content and the HDPE matrix stress state around the voids. At higher % Al_2_O_3,_ the interparticle distance increases make matrix hard to undergo yielding, decreasing the impact strength. When the Al_2_O_3_ content was less than 5% weight, the interparticle distance reaches a minimum which can change into plastic yield to improve the impact strength. Lesser % Al_2_O_3_ decreases the interparticle distance which makes HPMC behave like brittle.

### 3.2. Tribological Properties

**Figure 10:**
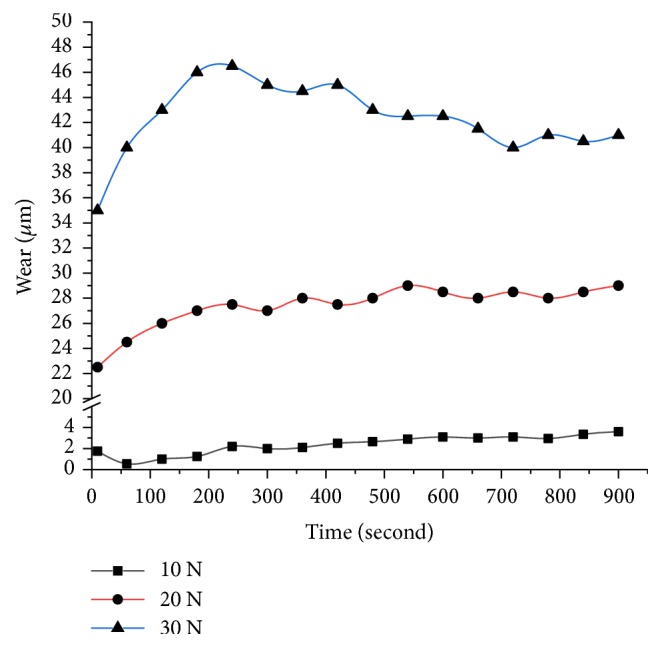
Wear varying with contact time at various loads and % reinforcements.

**Figure 11:**
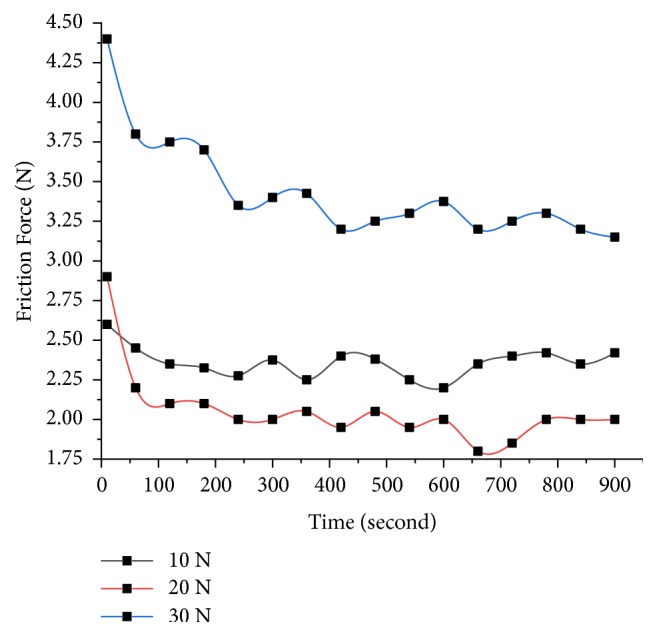
Frictional force varying with time of contact for 20% Al_2_O_3_.

**Figure 12:**
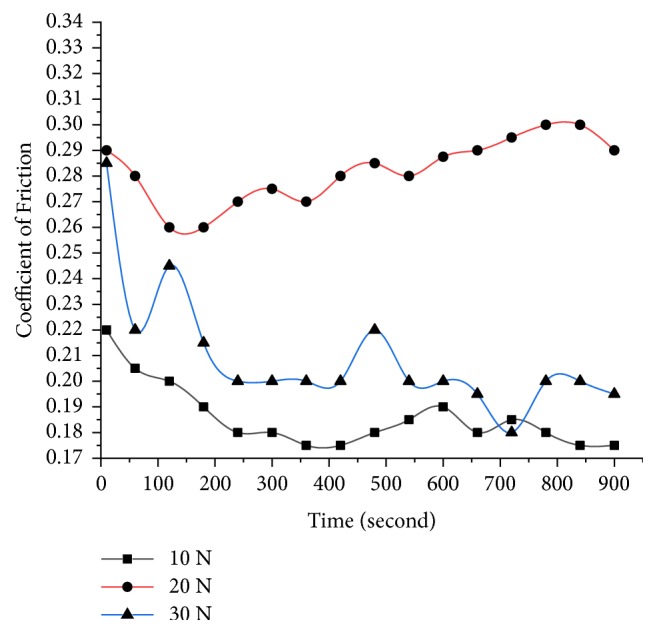
Variation of coefficient of friction at various loads and for 20% Al_2_O_3_.

The wear loss regarding height measurement using a pin-on-disc tester is taken down under different loads (10 N, 20 N, and 30 N) at a constant speed of 500 rpm for different pin specimens prepared with an ambient temperature of 20±1°C and a relative humidity of greater than 40±5% RH. The various observations were made in the wear analysis test. Firstly, the increase of the load on the specimen and sliding time increases the wear loss but decreases with the increase of % of alumina at fixed 10% of TiO_2_ as shown in Figure 10. Secondly, the alumina particles which are solid offer resistance to wear as long as bonding is maintained and uniform dispersion is achieved. Finally, frictional force decreases with the increase of alumina particles as they bear higher load without loss of weight or grain size and in turn the coefficient of friction also decreases as shown in Figures 11 and 12.

## 4. Conclusions

In the present study, the various observations are drawn based on the investigations conducted on hybrid polymer matrix composites (polymeric biocomposite) for orthopaedic applications. There are a variety of applications with the human body as implantable materials for bone surgery especially alternate for hard and soft tissues, bone cement, grafting, fixation plates, and hip joint replacement. With the increasing percentage of Al_2_O_3_ in the HPMC, the tensile, flexural strength, and hardness increased. Density has improved with reinforcing particles. Impact strength, coefficient of friction, frictional force, and wear decrease with an increase in the alumina percentage in the composite material. SEM image analysis has shown a homogeneous distribution of reinforcing particles with proper bonding between matrix and reinforcement without moulding imperfections. 70% HDPE/10% TiO2 /20% Al_2_O_3_ combination produces suitable biomaterial in orthopaedic applications during the processing of various tests. No corrosion was observed in the samples after a period of 48 hours at a pH value of 7. Injection moulding process is a successful fabrication technology for preparing biomaterial without any casting defects.

## Figures and Tables

**Table 1:** Composition of composites prepared.

**Sample No.**	**HDPE in weight **%	**TiO**_**2 **_ **in weight **%	**Al**_**2**_ **O**_**3**_ ** in weight **%
1	85	10	05

2	80	10	10

3	75	10	15

4	70	10	20

**Table 2:** Comparison of yield strength, strain at failure, and elongation of HPMC samples.

**Sample**	**Yield strength (MPa)**	**Strain at Failure (**%**)**	**Elongation (mm)**
**01**	**28.5**	**3.5**	**4.2**
**02**	**30.2**	**3.8**	**4**
**03**	**31.25**	**4**	**3.9**
**04**	**33.355**	**4.7**	**3.75**

**Table 3:** Corrosion test results.

**S. No.**	%** of** Al_2_O_3_	**Time in Hours**	**Observation**
01	5	24	No corrosion was observed
02	10	24	No corrosion was observed
03	15	24	No corrosion was observed
04	20	24	No corrosion was observed

## Data Availability

The data analyzed for this manuscript is our research work and is available for public use.
